# F198S Gerstmann-Sträussler-Scheinker Syndrome With Parkinsonism, Dyskinesia, and Abnormal (I-123)-FP-CIT Single-Photon Emission Computed Tomography: A Case Report

**DOI:** 10.7759/cureus.50594

**Published:** 2023-12-15

**Authors:** Rena Y Jiang, Stephen Aradi

**Affiliations:** 1 Neurology, University of South Florida (USF) Health Morsani College of Medicine, Tampa, USA; 2 Neurology, Carol & Frank Morsani Center for Advanced Healthcare, Tampa, USA

**Keywords:** movement disorder, dat-spect, gerstmann-straussler-scheinker syndrome, prion, case report

## Abstract

Gerstmann-Sträussler-Scheinker syndrome (GSS) is an autosomal dominant neurodegenerative disease caused by point mutations in the prion protein gene (PRNP)*.* While variable, the clinical presentation typically encompasses progressive cerebellar ataxia, pyramidal signs, and cognitive impairment. Here, we report a case of F198S-associated GSS manifesting levodopa-responsive parkinsonism, levodopa-induced dyskinesia, and an abnormal (I-123)-FP-CIT single-photon emission computed tomography (DaT-SPECT).

A 66-year-old male patient presented with six years of progressive recall and language impairment, with an initial impression of primary progressive aphasia. Over time he developed progressive cerebellar ataxia and akinetic parkinsonism. There was a family history of ataxia in multiple family members. Levodopa was prescribed up to 450 mg per day without benefit. Genetic testing at age 69 revealed a heterozygous F198S mutation in the PRNP gene, with MV heterozygosity at codon 129. At age 70, he developed mild generalized choreiform dyskinesia. Levodopa was discontinued, resulting in the resolution of dyskinesia with a concomitant marked worsening of akinetic parkinsonism. DaT-SPECT demonstrated bilaterally reduced putaminal binding.

This case highlights that GSS can resemble atypical parkinsonism both clinically and with DaT-SPECT imaging. Taking a salient family history and other clinical features into consideration, GSS should be added to the differential diagnoses of such patients.

## Introduction

Gerstmann-Sträussler-Scheinker syndrome (GSS) is an autosomal dominant neurodegenerative prion disease characterized by the deposition of prion protein (PrP) immunopositive amyloid plaques in cerebral and cerebellar parenchyma. There are at least 19 identified missense mutations in the PRNP gene that present with a GSS-like phenotype. In addition, the genotype at codon 129 of PRNP-commonly methionine or valine may alter the clinical characteristics of the disease [1].

Pathohistology of GSS reveals cerebral hemisphere and cerebellar vermis atrophy. PrP amyloid deposits-which are the protein product of PRNP-are found in various brain regions including the cerebral cortex, basal ganglia, and cerebellum. Neurofibrillary tau tangles with a paired helical structure, similar to those seen in Alzheimer’s disease, may also be seen. Spongiform changes of the brain are present in some cases but with lower prevalence than tau tangles [1].

The age of symptom onset usually ranges between the third and sixth decade of life. Symptoms may progress as rapidly as several months, or more slowly over the course of one to two decades [1]. Progressive cerebellar ataxia, pyramidal signs, and cognitive decline are the most common symptoms, though other features may co-occur and differ within families and carriers of the same mutations, including parkinsonism [1].

Two reported cases of D202N-related GSS demonstrated abnormal (I-123)-FP-CIT single-photon emission computed tomography (DaT-SPECT) imaging, but this imaging modality has not been reported in other genetic subtypes of GSS, including F198S. D202N usually presents around the eighth decade of life with axial akinetic rigidity, downgaze impairment, dementia, and hyperreflexia. F198S generally presents with a wider variability in age of onset. Eye movement abnormalities, memory problems, and psychotic depression are common initial manifestations. Here, we describe a case of F198S mutation GSS manifesting levodopa-responsive parkinsonism, levodopa-induced dyskinesia, and an abnormal DaT-SPECT. We demonstrate the importance of considering GSS in the workup of atypical parkinsonism with a strong family history.

Parts of this article were presented as a poster at the 2022 International Parkinson and Movement Disorder Society International Congress in Madrid, Spain.

## Case presentation

**Figure 1:**
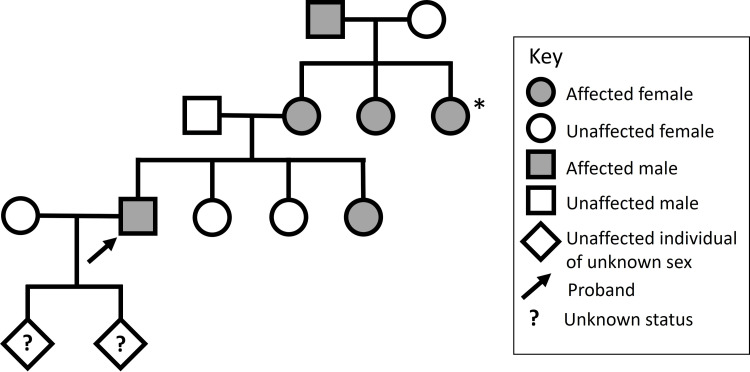
Pedigree * indicates the possibility of additional siblings not represented.

A 66-year-old right-hand dominant male with a past medical history of essential hypertension and developmental speech disorder presented with a six-year history of short-term memory loss and gait abnormalities. He reported mild dysphagia and shaky handwriting. His partner reported that his previously outgoing nature had become reserved, apathetic, and anxious. He had no history of smoking, alcohol use, or drug use, and no dream enactment or hyposmia. His family history was positive for a phenotype encompassing progressive parkinsonism, cerebellar ataxia, and cognitive impairment in his maternal grandfather, two maternal aunts, mother, and sister. He also had two unaffected sisters and two children of unknown status (Figure 1). Initially, the examination was notable for progressive expressive language deficits with effortful speech and multidomain mild cognitive impairment without frank cerebellar ataxia or parkinsonism. On detailed neuropsychological testing, difficulties were noted in multiple cognitive domains including select aspects of learning and memory (verbal word list learning, working memory); language (verbal associative fluency, spelling, comprehension for complex commands); and tasks dependent upon psychomotor speed and dexterity. Compared to his prior exams, the patient’s memory had been stable; repetition and naming had improved; and cognitive flexibility had improved (although improvement on this latter task may be due to practice effects). The decline was found with verbal associative fluency and probably occurred with spelling. The pattern of performance was consistent with a disruption of functions of predominantly left frontal-temporal or left subcortical regions (basal ganglia, thalamus). While primary progressive aphasia remained in the differential diagnosis, subcortical dementia, or early senile dementia of Alzheimer's type was also considered. 

Magnetic resonance imaging (MRI) performed at an outside institution reportedly demonstrated mild diffuse atrophy and mild periventricular white matter disease, though images were unavailable for direct review for our evaluation.

Over time, he developed progressive cerebellar gait, appendicular ataxia, and bradykinesia. A trial of carbidopa/levodopa 25/100 mg, up to one-and-a-half tablets three times per day, did not provide a clear motor benefit or emergence of dyskinesia. An Athena dominant ataxia panel did not identify any pathogenic abnormalities in the tested genes. Subsequent whole exome sequencing identified a pathogenic, heterozygous c.593T>C mutation (F198S) in the PRNP gene, with MV heterozygosity at codon 129.

At age 71, the patient exhibited supranuclear gaze palsy. Specifically, there were saccadic intrusions consisting of square wave jerks. Extra-ocular movements were notable for saccadic pursuits and restricted vertical gaze upward and downward. Vertical gaze was intact with oculocephalic reflex testing. Saccade generation on optokinetic nystagmus testing was impaired both horizontally and vertically. There was no nystagmus.

The patient also exhibited generalized chorea, asymmetric irregular jerky rest tremor, and multifocal and action-induced myoclonus. Levodopa was discontinued given the emergence of bothersome chorea and the apparent lack of significant motor benefit. Two weeks after discontinuation of levodopa, chorea was absent but his akinesia and gait had worsened. After reinitiation of levodopa at 100 mg three times per day, his akinesia improved and his choreiform dyskinesia returned.

**Figure 2:**
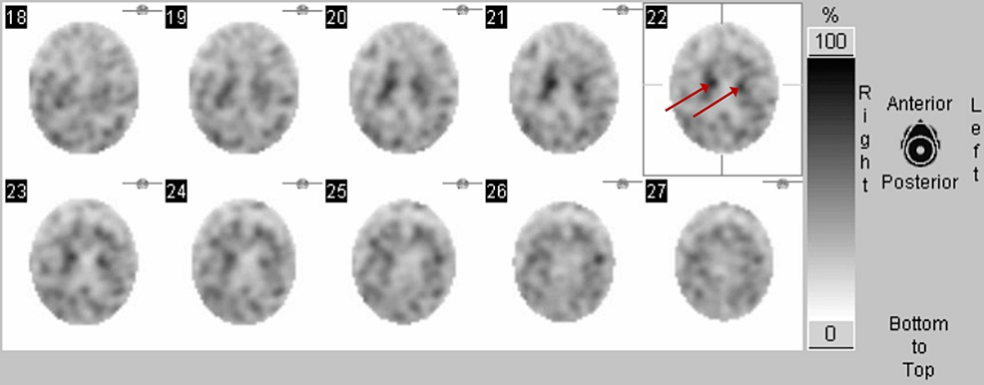
Axial images of (I-123)-FP-CIT DaT-SPECT Images show bilaterally absent putamen binding and heterogenous reduced binding in the left caudate nucleus more than the right. Red arrows indicate bilateral caudate nuclei. DaT-SPECT: (I-123)-FP-CIT single-photon emission computed tomography

At this stage, DaT-SPECT revealed absent binding in the bilateral putamina and markedly reduced binding in the left caudate nucleus more than in the right caudate nucleus (Figure 2).

## Discussion

Of the approximately 390 cases of GSS reported to date, the third most prevalent mutation is F198S-129V. Patients often present with clumsiness, ataxia, dysarthria, parkinsonism, short-term memory loss, and cognitive impairment. Early-stage MRI may show cerebellar atrophy. VV homozygosity at codon 129 is associated with an up to 10 year earlier age at onset compared to MV heterozygosity. Pathohistology reveals PrP amyloid, PrP diffuse plaques, and severe tau neurofibrillary pathology. PrP has been found in the stratum lacunosum-moleculare, hippocampal CA1, and subiculum-which are key regions for encoding memory. PrP has also been found in frontal, insular, temporal, and parietal cortices. In some cases, the neocortex has been found to contain alpha-synuclein immunopositive Lewy bodies. Interestingly, spongiform changes have not been noted in analyzed cases of F198S [1].

The abnormal DaT-SPECT in the present case may be consistent with previously reported PrP and tau accumulation in the caudate nucleus and putamen of patients with GSS [1]. To our knowledge, however, there is no consistent histopathological evidence of an isolated presynaptic dopamine deficit in GSS that could explain the patient’s response to levodopa.

There have been two previously reported cases of D202N-related GSS that demonstrate abnormal DaT-SPECT (Table 1). Like our patient, the case reported by Plate et al. 2013 [2] had levodopa-responsive parkinsonism and memory loss. However, this patient had daytime sleep attacks and fluctuating cognitive dysfunction resembling dementia with Lewy bodies, and the presence or absence of treatment-related dyskinesia was not reported. The case reported by Baiardi et al. 2020 [3] displayed similar clinical features as our patient as memory loss and ataxia-but levodopa response was not reported.

**Table 1:** DaT-SPECT results and select variables in prion disease cases GSS: Gerstmann-Sträussler-Scheinker; CJD: Creutzfeldt-Jakob disease; DaT-SPECT: (I-123)-FP-CIT single-photon emission computed tomography, MM: Methionine homozygosity; VV: Valine homozygosity; MV: Methionine-valine heterozygosity

No.	Reported diagnosis	PRNP mutation; codon 129	Dopa-responsive	Treatment-induced dyskinesia	DaT-SPECT result	Reference
1	GSS	D202N; VV	Yes	Not reported	Abnormal; right > left putamina	Plate et al. (2013) [2]
2	GSS	D202N; VV	Not reported	Not reported	Abnormal; right caudate and bilateral putamina	Baiardi et al. (2020) [3]
3	Familial CJD	A117V	No	Not reported	Abnormal; bilateral basal ganglia	Malek et al. (2017) [4]
4	Familial CJD	V180I; MM	No	No	Abnormal; bilateral putamina	Tomizawa et al. (2020) [5]
5	Sporadic CJD	Not reported	Not reported	Not reported	Abnormal; left > right putamina	Tang et al. (2022) [6]
6	Sporadic CJD	None; MV	Not reported	Not reported	Abnormal; right > left heterogenous uptake	Tilley et al. (2019) [7]
7	Sporadic CJD	None; MV	Not reported	Not reported	Abnormal; left > right putamina and left caudate	De León et al. (2018) [8]
8	Sporadic CJD	None; MM	Not reported	Not reported	Abnormal; left > right putamina	Ragno et al. (2009) [9]
9	Variant CJD	Not reported	Not reported	Not reported	Abnormal; bilateral caudate and right putamen	Magnin et al. (2011) [10]
10	Sporadic CJD	Not reported	Not reported	Not reported	Abnormal; bilateral caudate	Renard et al. (2010) [11]
11	Sporadic CJD	Not reported	Not reported	Not reported	Normal	Park et al. (2019) [12]
12	Sporadic CJD	None; MV	Not reported	Not reported	Abnormal; right caudate tail	Kim et al. (2014) [13]
13	Sporadic CJD	Not reported	No	Not reported	Abnormal; bilateral putamina and left caudate	Rissanen et al. (2014) [14]
14	Sporadic CJD	Not reported	Not reported	Not reported	Normal	Lee et al. (2022) [15]
15	Sporadic CJD	Not reported	Not reported	Not reported	Normal	Lee et al. (2022) [15]

With the limited reports of DaT-SPECT results in GSS cases, we also examined other prion diseases, identifying 13 cases of reported Creutzfeldt-Jakob disease (CJD) (Table 1). Two cases of CJD were familial, one was variant, and the remainder of the 13 were sporadic. All except three cases of sporadic CJD showed abnormal DaT-SPECT. Both cases of familial CJD and one case of sporadic CJD were assessed for levodopa responsiveness, but all three yielded negative results.

There are several limitations of the present case. First, clinical information is not available for the patient’s relatives who had a similar presentation. Assessing levodopa response and obtaining DaT-SPECT results could suggest the prevalence or consistency of the present findings in F198S GSS cases. Additionally, cutaneous biopsy for phosphorylated alpha-synuclein could have been considered to rule out an unlikely concomitant synucleinopathy. More quantitative measures of clinical improvement in parkinsonism, such as Unified Parkinson’s Disease Rating Scale motor scores, could have also been collected.

## Conclusions

To our knowledge, this is the first reported case of a patient with GSS that manifests a complement of abnormal DaT-SPECT, levodopa-responsive parkinsonism, and levodopa-induced dyskinesia. It is also the first report of DaT-SPECT imaging in F198S GSS. GSS is likely to be underdiagnosed due to its rarity and variable clinical presentations. This case highlights the idea that GSS can resemble atypical parkinsonism both clinically and with imaging. Taking a salient family history and other clinical features into consideration, GSS should be added to the differential diagnoses of such patients.
